# The Association Between Dietary Intake and Improvement of LARS Among Rectal Cancer Patients After Sphincter-Saving Surgery-A Descriptive Cohort Study

**DOI:** 10.3389/fsurg.2022.892452

**Published:** 2022-05-18

**Authors:** Wen Liu, Hai Ou Xia

**Affiliations:** ^1^ School of Nursing and Health Management, Shanghai University of Medicine & Health Sciences, Shanghai, China; ^2^ School of Nursing, Fudan University, Shanghai, China

**Keywords:** diet, sphincter-saving surgery, rectal cancer, anterior resection syndrome, nutrient

## Abstract

**Background:**

Dietary management was an important strategy for controlling low anterior resection syndrome (LARS) after sphincter-saving surgery, however, the influencing dietary factors of LARS are not completely clear. This study aims at exploring the specific association between perioperative intake of foods and nutrients and the improvement of LARS within the first 6 months after surgery.

**Methods:**

This study applied a prospective cohort design. 210 consecutive patients were admitted in the colorectal surgical ward after the sphincter-saving surgery in a tertiary hospital in China from May to November in 2020. The perioperative food intake was assessed by the food frequency questionnaire, and the bowel symptoms were assessed by the Low Anterior Resection Syndrome Score Scale. The binary logistic regression was used to analyze the collected data.

**Results:**

It was found out that both the intake of oil before surgery and at 6 months after surgery were significantly associated with the improvement of LARS. The average daily intake of livestock and poultry meats and oil during the first 6 months after sphincter-saving surgery were also associated with the improvement of LARS.

**Conclusions:**

The relationship between the intake of Livestock and poultry meats and oil and the improvement of LARS was significant in this study. It provides evidence for medical staff to make up effective interventions of moderating diet to promote the relief of LARS during the first 6 months after sphincter-saving surgery.

## Introduction

Colorectal cancer is one of the most prevalent malignant tumors worldwide (1). Colorectal cancer ranked third in incidence and second in mortality among all types of cancers in 2020 (1). In recent years, due to changes in the lifestyle and dietary patterns of Chinese residents, the incidence of colorectal cancer(CRC) has increased annually, and the incidence of rectal cancer is higher than that of colon cancer (2, 3).

A variety of surgical approaches are used to treat primary rectal cancer lesions, which include local procedures (e.g., polypectomy, transanal local excision, and transanal endoscopic microsurgery [TEM]), and more invasive procedures involving transabdominal resection (e.g., low anterior resection [LAR], proctectomy with total mesorectal excision [TME] and coloanal anastomosis, abdominoperineal resection [APR]) (4). With the rapid development of surgery technology, sphincter-saving surgery has been widely used, and it has been reported that 62%–85% of patients with rectal cancer undergo sphincter-saving surgery (5).

However, saving the anus does not mean saving its function. Different bowel symptoms have emerged following sphincter-saving surgery, including fecal incontinence, urgent and frequent defecation and bowel movements at night. All of these symptoms are collectively called low anterior resection syndrome (LARS), leading to a severe influence on the quality of life of patients after sphincter-saving surgery. Many studies have evaluated the prevalence and severity of LARS after sphincter-saving surgery; among these studies the prevalence of LARS ranged from 55% to 91.6%, while the percentage of severe LARS ranged from 29% to 58.3% (6–10). Although LARS improved over time after surgery, it is also reported in some prospective studies that the effect of LARS on the quality of life could exist up to more than 10 years after the sphincter-saving surgery, which means that it may be permanent (11, 12).

Although some treatments for relieving LARS have been discussed in previous studies, LARS treatment still carries difficulties because of a lack of well-conducted, randomized multicenter trials (13). The standard treatment available to deal with LARS is currently scarce, and self-management is reported to be the main method to control LARS after sphincter-saving surgery, in which dietary modification after surgery has played an irreplaceable role in controlling bowel symptoms. More than 96% of patients reported a change in diet to control bowel symptoms after surgery (14). Diet could influence defecation dysfunction because different dietary components could change intestinal motility, moderate gut microbiota and alter fecal morphology (15).

Research on the effects of specific foods and nutrients on defecation dysfunction after sphincter-saving surgery is still scarce. Existing studies have mainly focused on exploring patients’ subjective experiences after sphincter-saving surgery to examine the relationship between dietary factors and LARS. Yin et al. reported that 66% of patients reflected the close relationship between a specific food and bowel symptoms (14). In another study, diet self-management was also the most common behavior to control LARS (16). Some specific foods were reported in these limited studies to aggravate or relieve LARS. Greasy foods and dairy products were pointed out in some studies that could aggravate diarrhea symptoms (14, 17). A qualitative study showed that patients with LARS needed to avoid taking cereals, beans, nuts, popcorns, lettuces and onions; and foods suitable for defecation were liquefied foods (except for soft drinks) and high-fiber food (17). High-fiber foods have also been found in some other studies to be beneficial for managing fecal incontinence symptom (15, 18). Given the results of these limited studies, the relationships between some specific foods and nutrients and bowel symptoms were not consistent among different studies, which need further identification in future studies.

Although some studies have noticed the associations between dietary intake and LARS after sphincter-saving surgery, most patients in these studies tried to change their dietary intake to manage defecation dysfunction through trial and error, and the existing dietary modifications are lack of scientific guidance. It is noteworthy that there is still a shortage of research focusing on the relationship between specific food components and LARS; moreover, there is also a shortage of research discussing the long-term effect of dietary intake on LARS after sphincter-saving surgery.

Given the current research status, this study aimed to investigate the dietary intake and incidence of LARS in patients with rectal cancer after sphincter-saving surgery and to determine the associations of the intake of specific types and components of food and the improvement of LARS at different time points after sphincter-saving surgery. This information could provide cues for determining scientific dietary interventions to relieve LARS and to improve the quality of life of patients after sphincter-saving surgery.

## Methods

### Study Design

A prospective descriptive cohort design was applied in this study.

### Setting and Participants

Patients with rectal cancer after sphincter-saving surgery were recruited from a tertiary hospital in East China from May to November in 2020.

Participants recruited in this study included patients diagnosed with primary rectal cancer by pathology who had recently underwent sphincter-saving surgery and volunteered to participate in this study with informed consent. Patients who had hearing or cognitive disorders, suffered from complications (anastomotic fistula, rectovaginal fistula, etc.) after surgery, suffered from intestinal problems such as inflammatory bowel disease or irritable bowel syndrome before surgery or took some medicine like loperamide that might influence bowel function were excluded from this study. Patients with incomplete medical records were also excluded.

The sample size calculation formula for the descriptive correlation study was used to calculate the minimal sample size for this study, that is sample size = 4{(*μ_α_* + *μ_β_*)/ln(1 + *ρ*)/(1 − *ρ*)}^2^ + 3. According to the results of a pilot study focusing on the relationship between LARS and diet, *ρ* = 0.181–0.373.The calculation results showed that a sample of at least 72 patients was necessary, with a power of 80% and a level of significance of 0.05. Considering the attrition of participants, 210 participants were enrolled in the prospective cohort study.

The common clinical therapy plan for patients with rectal cancer in this study was implemented according to the Chinese Society of Clinical Oncology(CSCO) guidelines for colorectal cancer(version 2020) (19). Laparoscopic-assisted sphincter-saving surgery and robotic-assisted sphincter-saving surgery were the main methods for patients enrolled in this study. A fraction of patients needed to take radiotherapy combined with chemotherapy before surgery. In hospital, temporary colostomy has been put in the abdomen for some of rectal cancer patients taking the sphincter-saving surgery which would be closed about 6 months later. These patients were not enrolled in this study for the reason that because of the longitudinal cohort study design, the defecation status had been assessed for three times during the first 6 months following surgery. The temporary colostomy of these patients hadn’t been closed during the study process, thus the defecation function of remaining rectum and anus couldn’t be evaluated.

### Data Collection

Data related to perioperative dietary intake and LARS after sphincter-saving surgery were collected. Two nurses in ward were enrolled as investigators to assess patients. Each participant was contacted for the first time in the ward, and the baseline demographic data and clinical characteristics were obtained from medical records. Data collection was conducted in four timepoints. First, data related to preoperative dietary intake were collected in the hospital before the sphincter-saving surgery. In the prospective cohort study, every patient was followed up for 6 months at three time points: 6 weeks, 3 months and 6 months following sphincter-saving surgery. Data related to the postoperative dietary intake and the status of LARS after surgery were separately collected at these three timepoints after the sphincter-saving surgery. An interview was conducted at the outpatient visit at each time point after surgery to collect refined medical records if needed and data related to the postoperative dietary intake and the status of LARS after surgery.

### Research Tools

The FFQ questionnaire was applied to assess perioperative dietary intake. Patients’ postoperative status of LARS were measured with the Low Anterior Resection Syndrome Score (LARSS).

#### General Information Questionnaire

The general information questionnaire consisted of several items, including age, sex, employment status, education level, tobacco and alcohol use, and dietary habits before surgery.

#### Clinical Information Questionnaire

The clinical information questionnaire collected past medical information and data related to the treatment of rectal cancer, including the history of past chronic disease (diabetes, hypertension and cardiac disease) and surgery, history of personal and family cancer, history of past surgery, classification of tumor, length of bowel removed, anastomotic site, lymphatic metastasis, and adjunct therapy before and after surgery.

#### Low Anterior Resection Syndrome Score(LARSS)

This scale, developed by Emmertsen in 2012 to assess the bowel function of patients after sphincter-saving surgery, includes 5 items: (1) flatus incontinence; (2) shapeless stools and fecal incontinence; (3) frequency of defecation; (4) ability to control defecation; and (5) urgent defecation. The final score of this scale is the sum of the scores of all the items, with a total score of 0–42 points. The status of LARS can be divided into three levels: no LARS (0–20 points), minor LARS (21–29 points), and major LARS (30–42 points) (20). Minor LARS and major LARS are collectively defined as having LARS. The Chinese version of the LARSS was developed by Cao et al. and was applied in this study (21). Cronbach’s coefficient of the LARS score scale in this study was 0.920.

#### Food Frequency Questionnaire

Dietary intake was assessed with a 100-item quantitative food frequency questionnaire (FFQ), reflecting the consumption of the main food items by investigating the intake frequency of certain foods and the portion taken every time. The FFQ was designed by the Chinese Center of Disease Control and Prevention in 2010 to monitor people’s nutrition and dietary habits. More than 100 food items were assigned to 11 food categories. Intake frequency was estimated by using a scale of categories ranging from “times per day”, “times per week”, “times per month” and “times per year”. By multiplying the frequency and portion size, the average consumed amount was calculated and expressed as the intake in grams per day. Dietary intake analysis was performed by Nutrition Calculation Software, which was developed to calculate the consumption of different food categories and nutrients. The FFQ questionnaire was applied to survey the preoperative dietary intake and the postoperative dietary intake of 6 weeks, 3 months and 6 months after the sphincter-saving surgery.

### Statistical Analysis

**Figure 1:**
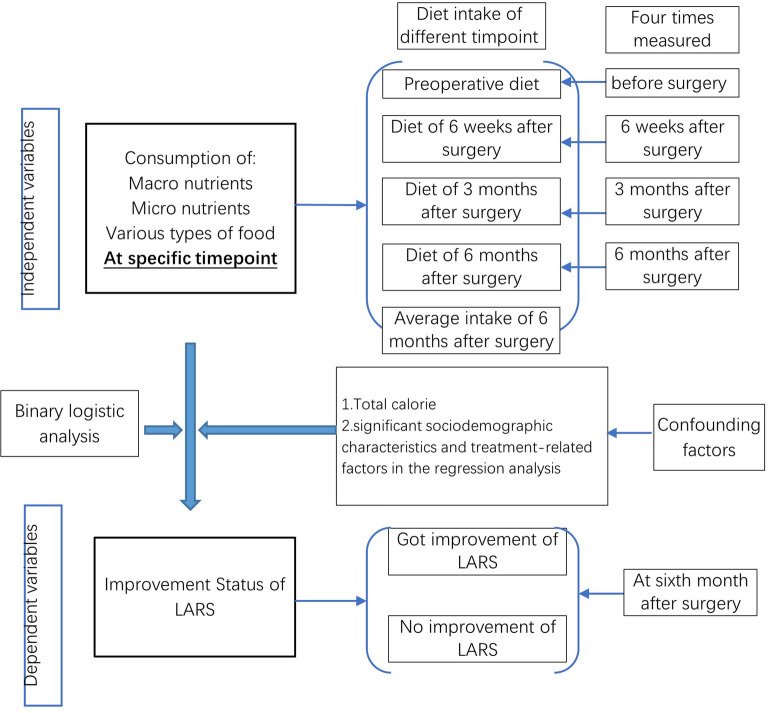
Analytic framework.

SPSS 21.0 was applied for the statistical analysis, and the statistical significance was set at *p* < .05 (2-tailed). Continuous variables are expressed as the mean (standard deviation), and categorical variables are described as numbers (%). The chi-square test or *Fisher’s* exact test was used to analyze counting data. *A t*-test was used for continuous variables when they were normally distributed, and the Mann-Whitney U test was used when they were not normally distributed. Binary logistic regression was used to analyze the associations of dietary intake at different time points and the improvement of LARS. The specific analytic framework is presented in Figure 1.

### Ethical Consideration

Ethical approval for this study was granted by the Institutional Review Boards of University, School of Nursing (#TYSQ2019-6-01)

## Results

### Participants’ Characteristics

In total, 210 patients who underwent sphincter-saving surgery finished the 6 months’ follow-up, and these data were included in this analysis .The process of follow-up is as follow:

Patients’ demographic, clinical, and surgical data are summarized in Table 1. The age range of the participants was 39–85 years, with a mean age of 61.3 years (SD = 9.833). The majority of them were male (63.3%), aged over 60 years(51.5%), and retired (54.8%). Only 45.7% of patients were in the normal range of weight. Most participants had not attended college to obtain a bachelor’s degree or higher (76.7%). A total of 28.1% of participants had a history of smoking, and 21.5% of participants had a history of drinking in the past 3 years. The vast majority of participants preferred to eat meat more than 2 times a week, to eat greasy or fried food more than once a week, to eat vegetables more than 4 times a week, and to eat whole-grain foods less than once a week.

**Table 1:** Single factor analysis of influencing factors of improvement status of LARS.

	*N*(%)	Improvement of LARS *N* = 76	No improvement of LARS *N* = 134
Sex			
Male	133 (63.3)	39 (51.3)	94 (70.1)
Female	77 (36.7)	37 (48.7)	40 (29.9)
Age (years)			
≤50	28 (13.3)	12 (15.8)	16 (11.9)
(50,60]	74 (35.2)	25 (32.9)	49 (36.5)
(60,70]	70 (33.3)	23 (30.2)	47 (35.1)
>70	38 (18.2)	16 (21.1)	22 (16.5)
BMI			
Normal	96 (45.7)	43 (56.6)	53 (39.6)
Overweight	96 (45.7)	31 (40.8)	65 (48.5)
Obesity	18 (9.6)	2 (2.6)	16 (11.9)
Occupation			
On-job	95 (45.2)	29 (38.2)	66 (49.3)
Retirement	115 (54.8)	47 (61.8)	68 (50.7)
Education			
Less than junior school	45 (21.4)	16 (21.1)	29 (21.6)
Middle and high school	116 (55.3)	43 (56.6)	73 (54.5)
College graduate and higher	49 (23.3)	17 (22.3)	32 (23.9)
Family economy			
Just enough	102 (48.6)	33 (43.4)	69 (51.5)
Rich	108 (51.4)	43 (56.6)	65 (48.5)
Smoke status before surgery			
No	151 (71.9)	61 (80.3)	90 (67.2)
Yes	59 (28.1)	15 (19.7)	44 (32.8)
Alcohol status before surgery			
No	167 (79.5)	68 (89.5)	99 (73.9)
Yes	43 (21.5)	8 (10.5)	35 (26.1)
Frequency of taking meat			
Less than one time a week	8 (3.8)	6 (7.9)	2 (1.5)
2–3 times a week	67 (31.9)	31 (40.8)	36 (26.9)
4–6 times a week	61 (29.1)	17 (22.4)	44 (32.8)
Everyday	74 (35.2)	22 (28.9)	52 (38.8)
Frequency of taking fried or greasy food			
Less than one time a week	170 (81.0)	62 (81.6)	108 (80.6)
More than one time a week	40 (19.0)	14 (18.4)	26 (19.4)
Frequency of taking sweet food			
Less than one time a week	157 (74.7)	56 (73.7)	101 (75.4)
2–3 times a week	27 (12.9)	8 (10.5)	19 (14.2)
More than 3 times a week	26 (12.4)	12 (15.8)	14 (10.4)
Frequency of taking sweet drink (like cococola)			
Less than one time a week	201 (95.7)	74 (97.4)	127 (94.8)
2–3 times a week	9 (4.3)	2 (2.6)	7 (5.2)
Frequency of taking vegetables			
Less than two times a week	21 (10)	10 (13.2)	11 (8.2)
4–6 times a week	91 (43.3)	37 (48.7)	54 (40.3)
Everyday	98 (46.7)	29 (38.1)	69 (51.5)
Frequency of taking whole-grain food			
Less than one time a week	153 (72.9)	52 (68.4)	101 (75.4)
2–3 times a week	37 (17.6)	16 (21.1)	21 (15.7)
More than 3 times a week	20 (9.5)	8 (10.5)	12 (8.9)
Frequency of taking pickled food			
Less than one time a week	173 (82.4)	67 (88.2)	106 (79.1)
More than one time a week	37 (17.6)	9 (11.8)	28 (20.9)
Frequency of taking too much food on weekend			
Less than one time a month	147 (70.0)	64 (84.2)	83 (61.9)
2–3 times a month	36 (16.2)	4 (5.3)	32 (23.9)
More than 3 times a week	27 (12.8)	8 (10.5)	19 (14.2)
Frequency of taking meals at restaurant			
Less than one time a month	145 (69.0)	59 (77.6)	86 (64.2)
Less than one time a week	27 (12.9)	8 (10.5)	19 (14.2)
2–4 times a week	21 (10.0)	7 (9.2)	14 (10.4)
More than 4 times a week	17 (8.1)	2 (2.7)	15 (11.2)
Distance between anal and the lower margin of tumor(cm)			
≤5	39 (18.5)	9 (11.8)	30 (22.4)
(6,10]	145 (69.1)	49 (64.5)	96 (71.6)
>10	26 (19.4)	18 (23.7)	8 (6.0)
Length of bowel being cut off(cm)			
(6,10]	97 (46.2)	39 (51.3)	58 (43.3)
>10	113 (53.8)	37 (48.7)	76 (56.7)
Cross-section diameter of bowel suffered from tumor			
One fourth of whole cross-section diameter	8 (3.8)	4 (5.3)	4 (3.0)
One third of whole cross-section diameter	27 (12.6)	13 (17.1)	14 (10.4)
Half of whole cross-section diameter	59 (28.2)	21 (27.6)	38 (28.4)
Two thirds of whole cross-section diameter	39 (18.7)	14 (18.4)	25 (18.6)
Three fourth of whole cross-section diameter	29 (13.8)	10 (13.2)	19 (14.2)
The whole cross-section diameter	48 (22.9)	14 (18.4)	34 (25.4)
Surgery type			
Laparoscopic	50 (23.8)	18 (23.7)	32 (23.9)
Robotic	160 (76.2)	58 (76.3)	102 (76.1)
Lymphatic metastasis			
No	127 (60.5)	42 (55.3)	85 (63.4)
Yes	83 (39.5)	34 (44.7)	49 (36.6)
Preoperative radiotherapy and chemotherapy			
No	185 (88.1)	67 (88.2)	118 (88.1)
Yes	25 (11.9)	9 (11.8)	16 (11.9)
Postoperative radiotherapy			
No	144 (68.6)	51 (67.1)	93 (69.4)
Yes	66 (31.4)	25 (32.9)	41 (30.6)
Postoperative chemotherapy			
No	26 (12.4)	9 (11.8)	17 (12.7)
Yes	184 (87.6)	67 (88.2)	117 (87.3)

Most participants underwent robotic anterior resection (76.2%). A total of 11.9% of them received chemotherapy and radiotherapy before surgery. The average distance between the anal and low margins of the tumor ranged from 3 to 15 centimeters, with a mean distance of 8.51 centimeters (SD = 2.97). The length of the bowel removed ranged from 6 to 19 centimeters, with a mean length of 11.30 centimeters (SD = 2.83) (Table 1).

### Improvement Condition of LARS After Sphincter-Saving Surgery

At 6 weeks, 3 months and 6 months after surgery, the percentages of participants suffering from LARS were 92.9%, 94.3% and 56.7%, respectively. A significant difference was noted among the percentages of LARS at different time points (*χ*^2^* = 53.238, P < 0.001*); according to the pairwise comparison, there was no significant difference between the percentages of LARS at 6 weeks and 3 months after sphincter-saving surgery (*χ^2 ^*= 0.082, *p *= 0.775); the percentage of LARS at 6 months after surgery was significantly lower than that at 3 months after surgery (*χ*^2^*^ ^*= 33.579, *p *< 0.001). Table 2 summarized the improvement condition of LARS.

**Table 2:** Status of LARS in different timepoints (*n* = 210).

	6 weeks	3 months	6 months	F/*χ*^2^	*p*
Occurrence status of LARS					
Non-occurrence of LARS *n* (%)	15 (7.1)	12 (5.7)	91 (43.3)	125.404	<0.001
Occurrence of LARS *n* (%)	195 (92.9)	198 (94.3)	119 (56.7)		
LARSS Score S(D)	31.7 (7.0)	31.1 (6.9)	22.4 (9.9)	48.890	<0.001

**Figure 2:**
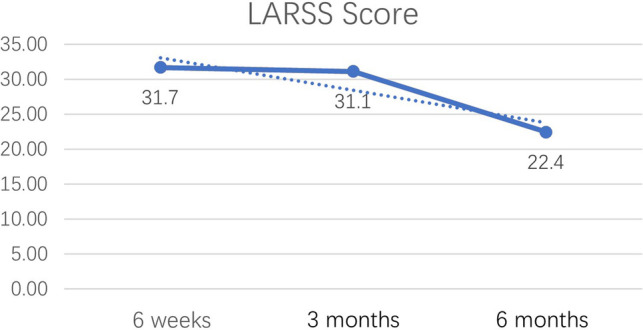
The changes of LARSS Score.

A significant difference was also noted among the scores of LARSS at different time points (F = 48.89, *P < 0.001*). As time went by, the score of LARSS had a tendency to decrease and the decreasing trend was most obvious at 6 months after the sphincter-saving surgery (Figure 2).

The improvement of LARS was defined as that from 6 weeks to 6 months after sphincter-saving surgery, and the status of LARS changed from “occurrence” to “non-occurrence”. Compared to the number of participants suffering from LARS at 6 weeks after surgery, 76 patients had complete improvement of LARS at 6 months after surgery with a status of LARS changing from occurrence to nonoccurrence. The rate of improvement of LARS at 6 months was 38.9% (Table 2).

### Analysis of General and Clinical Factors Influencing the Improvement of LARS

To determine the influence of sociodemographic characteristics and disease-related factors on the postoperative improvement of LARS, multivariate forward stepwise logistic regression was conducted (Table 3) The sociodemographic characteristics and disease-related factors were entered as the independent variables. The postoperative improvement of LARS was the dependent variable.

**Table 3:** Regression analysis of sociodemographic characteristics and treatment-related factors on postoperative improvement status of LARS.

	B	SE	Wald	*p*	*OR*	*OR* 95% CI
Alcohol consumption status before surgery	–1.281	0.601	4.311	0.034*	0.281	0.082–0.921
Distance between anal and tumor	1.120	0.478	6.991	0.008**	2.997	1.355–7.467
Radiotherapy status 3 months after surgery	–1.360	0.644	4.690	0.027*	0.251	0.068–0.881
Constant	–2.687	0.890	8.771	0.006**	0.071	

* *p < 0.05.*

** *p < *0.01.

The regression results showed that the alcohol consumption status before surgery (OR = 0.273, *p* = 0.036), the distance between anal and tumor (OR = 3.186, *p* = 0.008) and the radiotherapy status 3 months after surgery (OR = 0.256, *p* = 0.030)had a significant main effect on the postoperative improvement of LARS. The likelihood of getting improvement of LARS at the sixth month after sphincter-saving surgery among patients with the habit of taking alcohol before surgery decreased 71.9% compared to that of patient without the habit of taking alcohol before surgery. The likelihood of getting improvement of LARS at the sixth month after sphincter-saving surgery among patients with longer distance between anal and tumor was 2.997 times higher than that of patients with shorter distance between anal and tumor; The likelihood of getting improvement of LARS at the sixth month after sphincter-saving surgery among patients who was taking radiotherapy 3 months after surgery decreased 74.9% compared to that of patients without radiotherapy 3 months after surgery (Table 3).

### Analysis of Perioperative Dietary Influencing Factors on Postoperative Improvement of LARS

To determine the perioperative dietary factors influencing the postoperative improvement of LARS, multivariate logistic regression after adjusting for confounding factors was performed. The significant sociodemographic characteristics and average daily intake of different types of foods and nutrients were entered as independent variables (Table 4).

**Table 4:** Regression analysis of perioperative dietary factors on postoperative improvement status of LARS.

Dietary factors at different timepoints		B	S.E	*p*	*OR (95% CI)*
Before surgery	Intake of oil before surgery	−0.055	0.027	0.044*	0.947 (0.897,0.999)
	Alcohol consumption status before surgery	−1.401	0.625	0.025*	0.246 (0.072,0.839)
	Distance between anal and tumor	1.215	0.450	0.007**	3.372 (1.395,8.151)
	Constant	−1.188	1.137	0.296	0.305
6 weeks after surgery	Alcohol consumption status before surgery	−1.297	0.620	0.036*	0.273 (0.081,0.921)
	Distance between anal and tumor	1.159	0.434	0.008**	3.186 (1.361,7.462)
	Constant	−2.634	0.892	0.003	0.072
3 months after surgery	Intake of energy 3 months after surgery	−0.002	0.001	0.003**	0.998 (0.996,0.999)
	Radiotherapy status 3 months after surgery	−1.275	0.609	0.036*	0.279 (0.085.0.921)
	constant	2.355	0.873	0.007	10.536
6 months after surgery	Intake of oil 6 months after surgery	−0.065	0.030	0.032*	0.937 (0.883,0.994)
	Alcohol consumption status before surgery	−1.418	0.632	0.025*	0.242 (0.070,0.836)
	Distance between anal and tumor	1.320	0.463	0.004**	3.743 (1.511.9.271)
	Constant	−1.196	1.098	0.276	0.302

* *p < 0.05.*

** *p < 0.01 (multivariate logistic analysis have all been conducted after the adjustment of Alcohol consumption status before surgery status of radiotherapy 3 months after surgery, distance between the margin of anal and tumor, consumption of total calorie).*

The regression results showed that the daily intake of oil before surgery (OR = 0.947, *p* = 0.044) and the daily intake of oil 6 months after surgery (OR = 0.937, *p* = .032) had a significant main effect on the postoperative improvement of LARS. Patients with a higher rate of improvement in LARS after sphincter-saving surgery had a lower amount of daily intake of oil before surgery and 6 months after surgery (Table 4).

Multivariate logistic regression was conducted with the postoperative improvement of LARS as the dependent variable. The significant sociodemographic characteristics and average daily intake of different types of foods and nutrients among the 3 timepoints’ measurement within the first 6 months after sphincter-saving surgery were entered as independent variables. The results showed that the rate of improvement of LARS at 6 months among patients in the 1st tertile who consumed less than 33.10 g/day of livestock and poultry meat was 4.018 times higher than that of patients in the 3rd tertile who consumed more than 44.18 g/day of livestock and poultry meat (OR = 4.018, *p* = 0.015). There was no significant difference on the rate of improvement of LARS at 6 months between the intermediate intake of livestock and poultry meat tertile and the highest intake of livestock and poultry meat tertile of patients.(OR = 1.807, *p* = 0.306). It was also shown that the rate of LARS at 6 months among patients in the lowest intake of oil tertile, who consumed less than 26.67 g/day of oil, was 2.914 times higher than the patients in the highest intake of oil tertile, who consumed more than 30.01 g/day of oil (OR = 2.914, *p* = 0.047). There was no significant difference on the rate of improvement of LARS at 6 months between the intermediate intake of oil tertile and the highest intake of oil tertile of patients (OR = 0.828, *p* = 0.743) (Table 5).

**Table 5:** Regression analysis of average daily dietary intake within first 6 months after surgery on improvement status of LARS.

		B	S.E	*p*	OR (95% CI
Average intake of Livestock and poultry meats within the first 6 months after surgery	≤33.10 g/day	1.391	0.574	0.015*	4.018 (1.305,10.372)
	33.11–44.17 g/day	0.591	0.578	0.306	1.807 (0.582,5.607)
	≥44.18 g/day^a^			0.049	
Average intake of oil within the first 6 months after surgery	≤26.67 g/day	1.070	0.562	0.047*	2.914 (0.968,8.771)
	26.68–30 g/day	−0.189	0.576	0.743	0.828 (0.267,2.562)
	≥30.01 g/day^a^			0.052	
Distance between anal and tumor		1.433	0.475	0.003**	4.293 (1.443,10.192)
Constant		−4.483	1.173	0.000**	0.012

^a^ *Treated as a reference group. (multivariate logistic analysis have all been conducted after the adjustment of Alcohol consumption status before surgery status of radiotherapy 3 months after surgery, distance between the margin of anal and tumor, consumption of total calorie).*

* *p < 0.05.*

** *p < 0.01.*

## Discussion

In many previous studies, dietary intake was considered to have obvious effects on bowel symptoms after sphincter-saving surgery. However, the relationship between dietary intake and bowel symptoms was mostly discovered through qualitative methods exploring the subjective experiences of patients. Longitudinal prospective investigations of the improvement of LARS and dietary intake are still limited. In this study, the effects of perioperative dietary intake at different time points on the improvement of LARS were analyzed among patients after sphincter-saving surgery for the first time. The findings reported in this study related to the associations between dietary intake and the improvement of LARS make an important contribution to the understanding of how dietary intake influences the improvement of LARS after surgery.

In this analysis of a prospective cohort, some specific types of food affected the improvement of LARS, including the daily intake of oil and livestock and poultry meats. Meanwhile, some lifestyle habits before surgery were also shown to be important predictive factors influencing the improvement of LARS. The results reflected that alcohol consumption was an independent influencing factor of improvement status of LARS at 6 months postoperatively. Compared to patients without the habit of drinking before surgery, patients with alcohol consumption habits presented a lower probability of LARS improvement at 6 months after surgery. It has been reported that the main effects of alcohol on intestinal function are reducing the biodiversity of intestinal flora, affecting the normal movement of the gastrointestinal tract and changing intestinal mucosal permeability (22). Studies related to the alcohol consumption of patients with colorectal cancer have mainly focused on the relationship between alcohol consumption and the risk and outcome of colorectal cancer (23). However, there have been few studies on the effects of alcohol consumption before surgery on the occurrence and improvement of defecation dysfunction among patients after sphincter-saving surgery.

In this study, the average intake of oil before surgery was observed to be a predictive factor of the improvement of LARS within the first 6 months after surgery. The probability of the improvement status of LARS decreased 5.3% by every one-unit increase in the preoperative intake of oil. This finding provides evidence for medical staff to focus their attention on the association between preoperative dietary habits and the postoperative occurrence of defecation dysfunction. Strengthening the early assessment of patients’ dietary habits could identify the risk factors for LARS in a timely manner and provide evidence for treatments to manage LARS after surgery.

In addition, the effect of the intake of oil after surgery was noticeable, which significantly affected the improvement of LARS within the first 6 months after surgery. The probability of improving LARS decreased 6.3% by every one-unit increase in the intake of oil at 6 months after surgery. Oil in daily life includes animal oil and vegetable oil. On one hand, the effect of fats on defecation is reflected in the fact that vegetable oil could directly lubricate the intestines and that its decomposed product functions to irritate bowel movements (24). On the other hand, fats in food could strengthen bowel movements by binding to bile acids, making defecation more frequent and urgent (25).

Foods rich in oil were closely related to bowel symptoms after sphincter-saving surgery in some previous studies. In a qualitative study conducted by Sun et al., foods rich in fats were reported by patients to aggravate the symptoms of diarrhea after sphincter-saving surgery (17). Another study surveyed the self-management methods of LARS after surgery according to the relationship reported by patients in postoperative daily life, and dietary patterns of low fat and high fiber were chosen by 14.8% of patients to manage defecation dysfunction (26). In addition, fried foods and greasy foods were also reported in past studies that they were likely to worsen the fecal incontinence(27, 28). It could be seen that too much intake of oil in daily life after surgery is harmful to the management of LARS. According to the Chinese Dietary Guidelines(version 2016) (29), the recommended intake of oil is 25–30 grams per day. Based on the results presented in this study, patients with an intake of oil less than 26.67 g/day had a higher rate of improvement in LARS than patients with an intake of oil more than 30.01 g/day. To increase the improvement rate of LARS, this study recommends that patients with rectal cancer after sphincter-saving surgery be guided and monitored to limit their daily average intake of oil within the recommended intake range of Chinese Dietary Guidelines. Considering the study design and the representativeness and size of the sample in this study, the specific influencing mechanism of oil intake on the improvement of LARS, and the recommended range of intake of oil for patients after sphincter-saving surgery still need to be identified in future.

From the results of this study, the average intake of livestock and poultry meats within the first 6 months after surgery was also related to the improvement of LARS. Compared to patients eating less than 33.10 g/day of livestock and poultry meats, patients eating more than 44.18 g/ day of livestock and poultry meats showed a significantly lower probability of improvement of LARS at 6 months after surgery. The effect of the intake of livestock and poultry meats on bowel symptoms has been discussed in little research. A survey conducted by Martin J. reported that reducing the amount of daily intake of meats after sphincter-saving surgery was a method applied by many patients to control the occurrence of LARS themselves, indicating that too much intake of livestock and poultry leaded to severe bowel symptoms (30).The possible reason is that livestock and poultry are mostly rich in fat, which was reflected by some patients to have the function of aggravating bowel symptoms in previous qualitative study (31). This effects of fat on bowel symptoms can be also confirmed in other two studies which reported that dietary pattern of high fiber and low fat were chosen by the most patients to control bowel symptoms after sphincter-saving surgery (14, 26). Although it can be seen in these study that foods rich in fat may not good for defecation, the specific mechanism about how does intake of livestock and poultry influence the improvement of LARS is not clear now. Scientific evidence for the guidance of the intake of livestock and poultry meats after sphincter-saving surgery to manage LARS and enhance the improvement of LARS relies on further discussion and identification.

Some other foods, such as fruits, vegetables and milk products, showed some relationship with LARS in other studies conducted in some European countries. Several studies reported that too much intake of milk products may aggravate dirrhea (17, 27, 28). The specific effect of vegetables and fruits on LARS were not consistent in previous studies. In this study, the relationship between intake of fruits, vegetables and milk products and improvement of LARS was not significant. The differences among different studies maybe partly related to the different dietary culture between Chinese patients and patients in other countries, and meanwhile it maybe related to the different study instruments in these studies. More studies including much more study population maybe needed to conduct in future to further identify the specific relationship between intake of fruits, vegetables and milk products and the improvement of LARS.

This study is the first to report the effects of the intake of different types of foods and nutrients on the improvement of LARS during the first 6 months in a cohort of patients with rectal cancer after sphincter-saving surgery. The findings in this study reflected that the average daily intake of oil before surgery could predict the improvement of LARS after surgery to some extent. Keeping the average daily intake of oil and livestock and poultry meats within the certain range during the first 6 months after surgery could help with the improvement of LARS. Findings from this study provide some evidence for the management of LARS through moderating diet. Considering the scarce evidence to support the effect of the intake of different types of foods and nutrients on the improvement of LARS after surgery, future research is needed to focus on the causal relationship among specific food components and the occurrence of LARS; meanwhile, more studies exploring the mechanisms of the effects of different foods and nutrients on bowel symptoms are urgently needed.

### Limitations

It is acknowledged that the results of this study are limited to one surgical team at the same tertiary hospital with a small sample sizewhich may limit the generalization of the findings. Although some confounding factors were adjusted in the study, there might still be some other variables that were not fully considered, such as tumor size, the type of anastomosis and physical activity after surgery. During the first 6 months after surgery, some participants were missed to follow up for several reasons, such as failure to contact, loss of medical records and withdrawing midway through the study. Meanwhile, the effects of dietary intake on the improvement of LARS were only followed up until the 6 months after surgery, considering that it would take more than 6 months for LARS improvement for a portion of the patients; the long-term effects also need to be identified. Another limitation is the non-randomized approach applied in this study which makes the study findings should be taken with care.

## Data Availability

The raw data supporting the conclusions of this article will be made available by the authors, without undue reservation.
